# Estimation of the genetic susceptibility prevalence of primary ciliary dyskinesia via the gnomAD v4.1.0 database

**DOI:** 10.1186/s13023-026-04501-4

**Published:** 2026-07-20

**Authors:** Rutao Dai, Ya Dao, Xichen Zhang, Yongyu Ma, Xian Zhu, Yunshan Gao, Jun Wu, Liu Sun

**Affiliations:** 1 Affiliated Children’s Hospital of Kunming Medical University, Kunming Children’s Hospital, Kunming, 650100 China; 2 Department of Cardiothoracic Surgery, Kunming Children’s Hospital, Kunming, 650100 China; 3 Department of Computer Science, School of Engineering, Yuxi Normal University, Yuxi, 653100 China

**Keywords:** Primary ciliary dyskinesia (PCD), Carrier frequency, Genetic susceptibility prevalence, Genome aggregation database (gnomAD), Rare diseases

## Abstract

**Background:**

Primary ciliary dyskinesia (PCD) is a rare genetic disorder that is predominantly inherited in an autosomal recessive pattern and is caused by structural or functional ciliary abnormalities. Previous studies on the genetic susceptibility prevalence of PCD have been largely based on limited populations, and there is a lack of global estimates derived from large-scale population genetic databases. This study estimates the carrier frequency and genetic susceptibility prevalence of PCD via the latest data from the gnomAD v4.1.0 database and compares the results with those of previous studies.

**Methods:**

We selected genes related to PCD with “Definitive” or “Strong” associations as determined by the ClinGen Motile Ciliopathy Gene Curation Expert Panel. According to ACMG/AMP guidelines, variables were classified for pathogenicity, including loss-of-function variants (pLOFs) and pathogenic/likely pathogenic missense variants. Using the allele frequency (AF) of these variants from the Genome Aggregation Database (gnomAD) v4.1.0 (containing data from 807,162 individuals) and applying the Hardy‒Weinberg equilibrium principle, we calculated the global and population-specific genetic susceptibility prevalence and carrier frequency of PCD.

**Results:**

Among the 31 PCD-associated genes, 5252 eligible variants were included, comprising 5156 pLOF and 96 P/LP variants. The estimated global genetic susceptibility prevalence of PCD is approximately 1 in 20,103, with a corresponding overall carrier frequency of approximately 1 in 25. The Middle East region has the highest prevalence rate of 1 in 4,744, as well as the highest carrier frequency.

**Conclusion:**

The estimated global genetic susceptibility prevalence of primary ciliary dyskinesia is approximately 1 in 20,103, with an overall carrier frequency of 1 in 25, indicating significant heterogeneity among ethnic populations. The global genetic susceptibility prevalence estimate is lower than the previous estimate of 1/7,554.

**Supplementary Information:**

The online version contains supplementary material available at https://doi.org/10.1186/s13023-026-04501-4.

## Introduction

Primary ciliary dyskinesia (PCD) is a genetically heterogeneous disorder **predominantly** inherited in an autosomal recessive **pattern** [4, 19, 25]. The core pathophysiological mechanism involves structural or functional abnormalities of motile cilia, leading to impaired mucociliary clearance [28, 30]. The clinical manifestations are diverse, with the most common being chronic, recurrent respiratory infections, including neonatal respiratory distress, chronic sinusitis, otitis media, and bronchiectasis [3]. Approximately 50% of patients exhibit situs inversus totalis, known as Kartagener syndrome [31]. Additionally, male infertility is a common clinical feature [9]. Owing to the lack of specificity in clinical presentations, PCD is often misdiagnosed as asthma, allergic rhinitis, or immunodeficiency disorders, resulting in delayed diagnosis [17, 27].

In recent years, advancements in genetic sequencing technology have significantly enhanced the understanding of the genetic basis of PCD, making it one of the primary diagnostic tools for PCD [26]. Currently, approximately 65%-70% of PCD patients can have their pathogenic genes identified through genetic diagnosis [14]. The number of pathogenic genes associated with PCD continues to increase, from over 40 to more than 50 genes that have been identified [29]; these genes encode proteins involved in cilia assembly, structure, and function [5]. The ClinGen Motile Ciliopathy Gene Curation Expert Panel has systematically evaluated the strength of the association between these genes and disease, providing crucial evidence for molecular diagnosis [1]. Despite significant progress in genetic research on PCD, the true prevalence of PCD in the population remains unclear [8]. Previous genetic susceptibility prevalence estimates based on clinical registries or specific regional studies vary widely, ranging from 1/10,000 to 1/30,000 [6]. Sample size, inconsistent diagnostic criteria, and potential selection biases often limit these studies. The use of genetic variant data from large-scale population genomic databases (such as gnomAD) can provide a more direct estimation of genotype-based genetic susceptibility prevalence, offering a supplementary perspective for understanding disease burden. This study aimed to systematically assess the genetic susceptibility prevalence of PCD globally and across different ethnic groups via the latest gnomAD v4.1.0 database.

## Methods

### Database

**Fig. 1:**
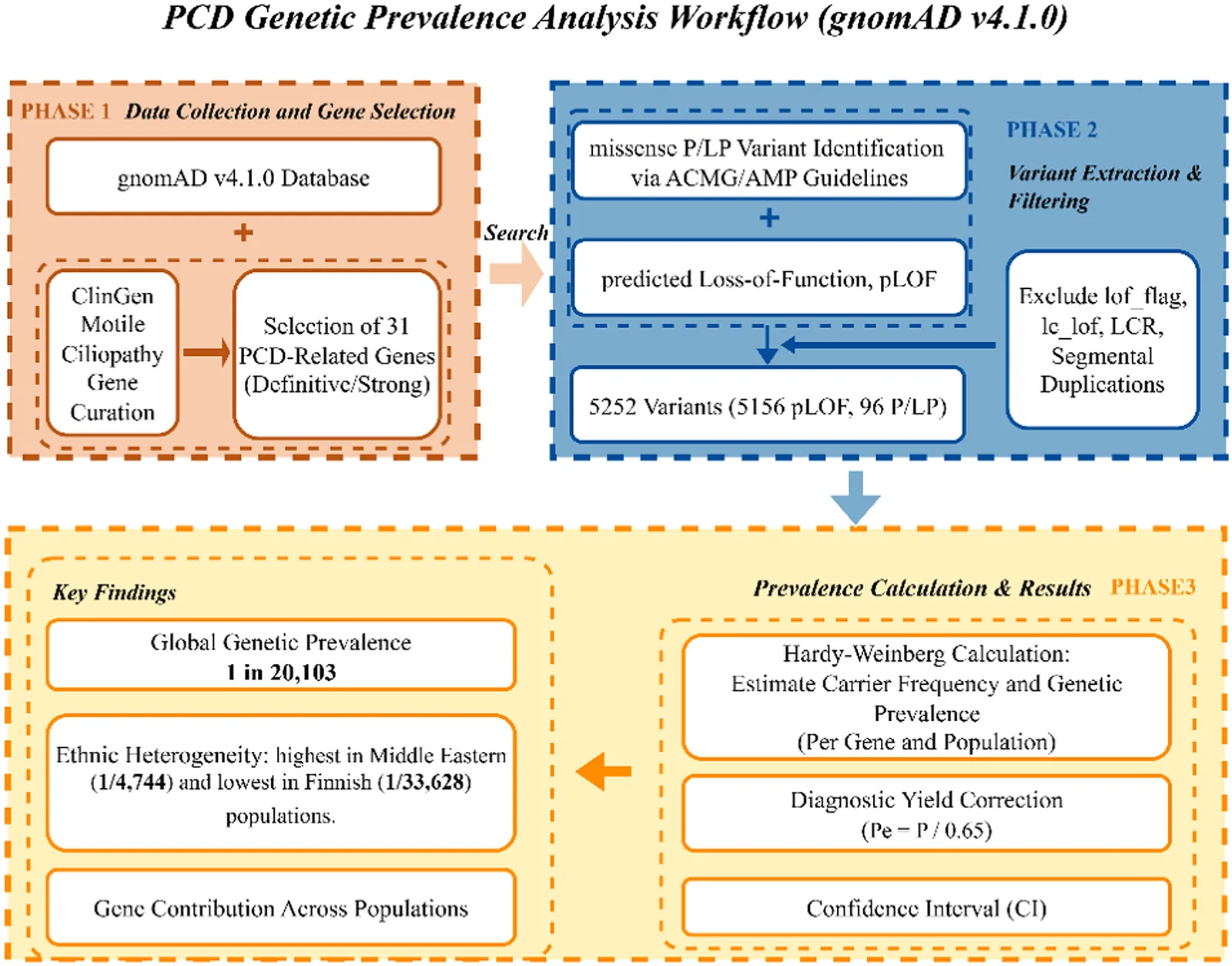
Flowchart for calculating genetic susceptibility prevalence

The method flowchart can be found in Fig. 1. This study utilized the publicly available genomic database gnomAD v4.1.0 (https://gnomad.broadinstitute.org/), and therefore, was exempt from institutional review board approval. The database integrates 730,947 whole exomes (416,555 individuals) and 76,215 genomes from various population genetic studies worldwide, totaling 807,162 individuals. These sequencing data are categorized into several major populations on the basis of inferred genetic ancestry, including African/African American (AFR), American Mixed/Latino (AMR), Ashkenazi Jewish (ASJ), East Asian (EAS), European (non-Finnish) (NFE), Finnish (FIN), Middle Eastern (MID), South Asian (SAS), and others (REMAINING) (Table 1). The gnomAD researchers implemented extensive quality control measures to remove poor-quality sequences and flag variants with questionable reliability [15]. This study primarily extracted information on predicted loss-of-function (pLoF) and missense variants in PCD-related genes from the database.

**Table 1:** gnomAD database composition according to population

Genetic Ancestry	Sample size
Africa/African American	37,545
Admixed American	30,019
Ashkenazi Jewish	14,804
East Asian	22,448
European (Finnish)	32,026
Middle Eastern	3031
European (non-Finnish)	590,031
Amish	456
South Asian	45,546
Remaining^*^	31,712
All	807,162

*There were 31,712 people with no assigned ethnicity according to gnomAD. The data are organized according to these terms, described as genetic ancestry. Notably, genetic ancestry is not comparable with race, ethnicity, or geographic identification. We used the genetic ancestry groupings described by the gnomAD investigators. More information about the ancestry assignment and population labels provided by different sequencing projects is available on the gnomAD website

### Gene selection

In accordance with the PCD pathogenic gene evaluation results released by the ClinGen Motile Ciliopathy Gene Curation Expert Panel, this study included all PCD-related genes located on autosomes whose association strength was rated as “Definitive” or “Strong” [26]. Thirty-one genes met the criteria, including *DNAH5*,* DNAI1*,* CCDC39*,* CCDC40*,* DNAH11*,* RSPH4A*, and *TP73*.

### Variant selection and filtering

Variants that may lead to loss of function (PLOF) and pathogenic/likely pathogenic missense variants were included. The pathogenicity interpretation of variants strictly followed the sequence variant interpretation standards and guidelines jointly issued by the American College of Medical Genetics and Genomics (ACMG) and the Association for Molecular Pathology (AMP) [23]. When applying these guidelines, we referred to the archived variants and their clinical significance classification information in the publicly available variant database ClinVar [18]. When multiple classifications existed for a variant in ClinVar, we included the variant only if there was consistent P/LP classification without conflicting interpretations (e.g., conflicting VUS or Benign calls). For variants with conflicts, we manually reviewed the supporting evidence; none met our inclusion criteria due to uncertainty, consistent with our conservative approach to minimize false positives. The stepwise variant filtering workflow is summarized in Supplementary Figure S1.

To ensure the accuracy and clinical relevance of variant annotation, this study prioritized the use of the standard transcript set (such as MANE Select) recommended by the MANE (Matched Annotation from NCBI and EMBL-EBI) project, which NCBI and EMBL-EBI jointly maintain for transcript selection [21].

On the basis of the above, we further established exclusion criteria to filter out potentially nonpathogenic variants or technical errors. Specifically, excluded variants include variants annotated as lof_flag (indicating possible false-positive LoF) or lc_lof (low-confidence LoF) in the gnomAD database and variants located in low complexity regions (LCR) or segmental duplication regions known to affect variant interpretation.

### Calculation of carrier frequency and genetic susceptibility prevalence [11–13]

PCD mainly follows an autosomal recessive inheritance pattern. For each screened P/LP variant ***i***, we obtained its allele frequency $\:{q}_{i}$ in the global population and major ethnic populations from the gnomAD v4.1.0 database. Assuming that the population conforms to the Hardy‒Weinberg equilibrium hypothesis, for each PCD-related gene (represented by ***G***), we first calculated the sum of the allele frequencies of all P/LP variants in the gene: $\:{q}_{G}=\sum\:_{i\in\:G}^{\:}{q}_{i}$. The disease incidence contribution of a single gene ***G*** can be estimated as$\:\:{P}_{G}={\left({q}_{G}\right)}^{2}$. The total genetic susceptibility prevalence (P) was calculated by summing the disease prevalence contributions of all independently analyzed genes $\:P=\sum\:{P}_{G}$. According to the HWE formula, the carrier frequency of a single gene ***G*** ($\:{C}_{G}$) can be calculated via the formula $\:{C}_{G}=2\times\:{q}_{G}\times\:\left(1-{q}_{G}\right)$. Since the allele frequency $\:{q}_{G}$ is usually small, $\:1-{q}_{G}$ is approximately * 1*. The carrier frequency of PCD was estimated by summing the carrier frequency contributions of all independent genes: $\:{C}_{PCD}=1-\prod\:_{G=1}^{n}(1-{C}_{G})$. Because approximately 65%-70% of currently diagnosed PCD patients have pathogenic genes identified earlier, the final disease prevalence calculation was adjusted accordingly, i.e., the estimated disease prevalence $\:{P}_{e}=P/0.65$. The 95% confidence interval (95% CI) of the disease incidence was calculated via the Wilson score interval, and all the above calculations were repeated at least twice to ensure accuracy.

## Results

**Fig. 2:**
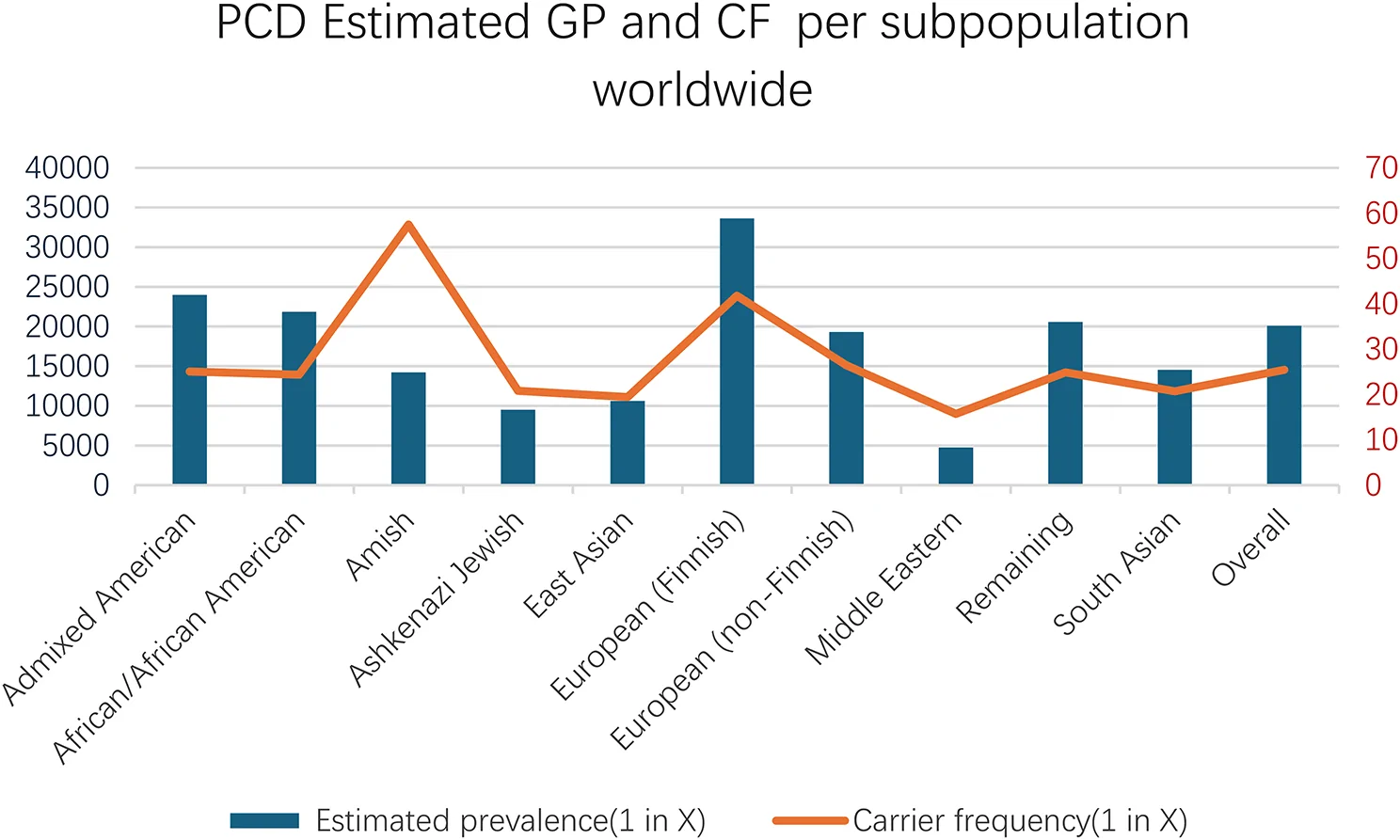
PCD estimated GP and CF per subpopulation worldwide

This study included a total of 31 pathogenic genes with “Definitive” or “Strong” associations with PCD. Through systematic screening of the gnomAD v4.1.0 database and pathogenicity interpretation on the basis of the ACMG/AMP guidelines, 5252 variants meeting the inclusion criteria were ultimately included. These variants comprised 5156 pLoF variants and 96 missense variants, with genes such as DNAH11, DNAH5, and DNAH9 carrying a relatively greater number of variants. By aggregating the allele frequencies of all identified variants, the estimated global genetic susceptibility prevalence (P) of PCD was approximately 1/20,103. There were significant differences in the estimated genetic susceptibility prevalence of PCD across different ethnic groups, with the highest prevalence observed in Middle Easterners (MIDs) at 1/4744 and the lowest in Finns at 1/33,628. Similarly, we further estimated the carrier frequency of PCD. The global overall carrier frequency of PCD is estimated to be as high as 1/25. Similar to genetic susceptibility prevalence, carrier frequency also shows significant differences among different ethnic groups, with the highest carrier frequency in Middle Eastern populations at approximately 1/16 and the lowest in the AMISH population at 1/57. (Table 2; Fig. 2)

Sensitivity analysis for the 65% diagnostic yield adjustment factor revealed that the adjusted global prevalence estimate ranges from 1/18,556 (using 60% yield) to 1/23,196 (using 75% yield) (Supplementary Table S1). The Middle Eastern population consistently showed the highest prevalence across all sensitivity scenarios (range: 1/4,378 to 1/5,472), while the Finnish population remained the lowest (range: 1/31,024 to 1/38,784). These results confirm that the observed population rankings and overall conclusions are robust to the choice of diagnostic yield parameter.

**Table 2:** PCD genetic susceptibility prevalence and carrier frequency in different ethnic groups

Genetic Ancestry	Unadjusted genetic prevalence* (q² sum) (95% CI)	(1 in X)	Adjusted prevalence^#^ (unadjusted / 0.65) (95% CI)	(1 in X)	Carrier frequency (1 in X)
Admixed American	2.71e-05 (4.12e-06–1.78e-04)	36,906	4.17e-05 (6.34e-06–2.74e-04)	23,988	25
African/African American	2.98e-05 (5.67e-06–1.56e-04)	33,606	4.58e-05 (8.72e-06–2.40e-04)	21,843	24
Amish	4.57e-05 (2.45e-07–8.44e-03)	21,886	7.03e-05 (3.77e-07–1.30e-02)	14,226	57
Ashkenazi Jewish	6.83e-05 (1.22e-05–3.83e-04)	14,633	1.05e-04 (1.87e-05–5.90e-04)	9512	21
East Asian	6.13e-05 (1.34e-05–2.80e-04)	16,321	9.42e-05 (2.06e-05–4.31e-04)	10,609	19
European (Finnish)	1.93e-05 (2.39e-06–1.56e-04)	51,736	2.97e-05 (3.68e-06–2.40e-04)	33,628	42
European (non-Finnish)	3.37e-05 (2.18e-05–5.21e-05)	29,715	5.18e-05 (3.35e-05–8.01e-05)	19,315	26
Middle Eastern	1.37e-04 (1.23e-05–1.52e-03)	7298	2.11e-04 (1.89e-05–2.34e-03)	4744	16
Remaining	3.16e-05 (5.52e-06–1.81e-04)	31,671	4.86e-05 (8.50e-06–2.78e-04)	20,586	25
South Asian	4.47e-05 (1.24e-05–1.61e-04)	22,378	6.87e-05 (1.90e-05–2.48e-04)	14,546	21
Overall	3.23e-05 (2.21e-05–4.73e-05)	30,927	4.97e-05 (3.40e-05–7.28e-05)	20,103	25

^*^The unadjusted prevalence represents the direct calculation from summed allele frequencies assuming complete detection of all pathogenic variants;

^#^the adjusted prevalence incorporates the 65% diagnostic yield to estimate total genetic prevalence including undiscovered variants

**Fig. 3:**
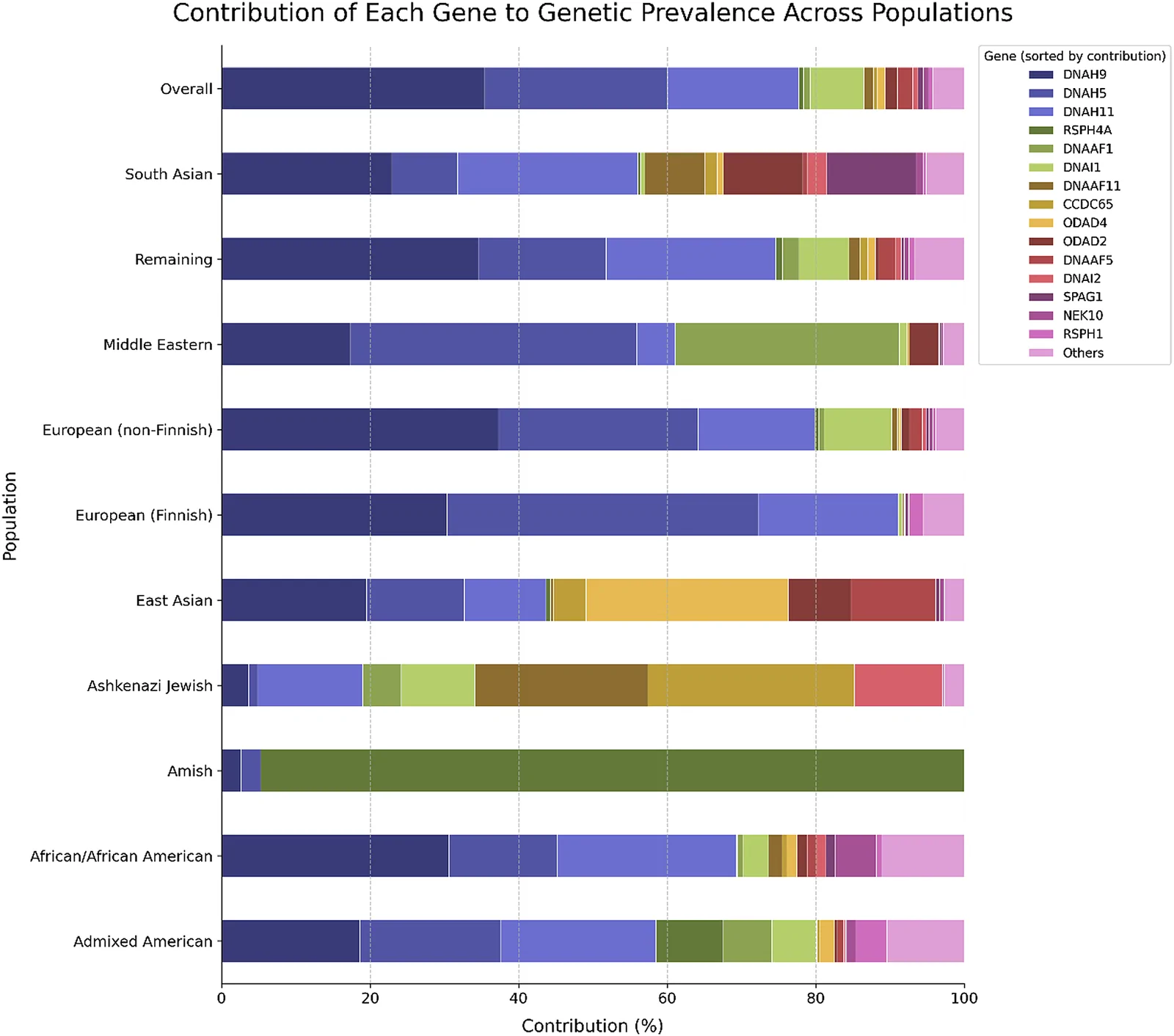
Relative contributions of the top PCD genes by population

Among the relative contributions of genes, the study conducted a comparative analysis of the relative pathogenic variant contributions of PCD-related genes across different global populations, revealing significant differences in the PCD pathogenic gene contribution profiles among various populations. In the overall population, pathogenic variants in the DNAH9 gene were the most common, accounting for approximately 35%. This was followed by *DNAH5* (approximately 25%) and *DNAH11* (approximately 18%). Notably, the *DNAH9*, *DNAH5*, and *DNAH11* genes accounted for the majority of the contributions in most populations. Other explicitly listed genes (*RSPH4A*, *CFAP300*, *DNAAF1*, *CCNO*, *DNAI1*, *ODAD4*, *ODAD2*, etc.) collectively contributed to approximately 22% of the cases. For details on the remaining populations, please refer to Fig. 3.

## Discussion

Primary ciliary dyskinesia (PCD) is a rare disorder predominantly inherited in an autosomal recessive pattern and is characterized by high heterogeneity in both genetic and clinical manifestations. Accurate assessment of its genetic susceptibility prevalence globally and among different ethnic populations is crucial for disease understanding, diagnostic strategies, resource allocation, and potential therapeutic development [12]. This study aims to systematically analyze variants in 31 genes closely associated with PCD pathogenesis, as evaluated by authoritative sources, using the largest current population genomic database, the Genome Aggregation Database (gnomAD) version 4.1.0. Through this analysis, we aim to provide the latest estimates of PCD genetic susceptibility prevalence and explore its distribution characteristics and potential influencing factors across different global populations.

It is important to distinguish between the ‘genetic susceptibility prevalence’ estimated in this study and true ‘clinical disease prevalence.’ Our estimate, derived from gnomAD v4.1.0—a database that intentionally excludes individuals with severe early-onset diseases—represents the lower bound of genetic susceptibility to PCD in the general adult population. Clinical disease prevalence, in contrast, reflects the proportion of individuals with manifest disease, which may be lower due to variable expressivity, incomplete penetrance, underdiagnosis, or premature mortality. Conversely, clinical prevalence could theoretically be higher than our estimate if environmental factors or modifier genes trigger disease in genetically susceptible individuals. For disease burden modeling, our minimal genetic prevalence (~ 1/20,000) provides a baseline for estimating the maximum potential impact of PCD if all genetically susceptible individuals developed disease, whereas clinical registry estimates (typically 1/10,000–1/30,000) reflect current real-world disease burden. The alignment between our estimate and the lower range of clinical estimates suggests that underdiagnosis remains a significant issue.

In this study, we identified and included 5252 eligible variants, comprising 5156 pLOF variants and 96 Pathogenic/Likely Pathogenic (P/LP) variants, all located within 31 genes with “Definitive” or “Strong” pathogenic associations with PCD (as per the ClinGen evaluation). Among these variants, pLOF variants predominated, which is consistent with the findings of previous studies [6]. On the basis of these variant data and the application of a 65% gene diagnostic rate correction factor, this study estimates the global adjusted genetic susceptibility prevalence of PCD to be approximately 1/20,103 (95% confidence interval: 13729–29435). Within the epidemiological conceptual framework, this “genetic susceptibility prevalence,” which is based on genotype frequency at birth, can functionally be regarded as the “incidence at birth” of the disease. The “incidence at birth” data provided by this study are crucial for future precise calculations of clinical prevalence and assessment of long-term societal disease burden by integrating survival curve data of PCD patients.

Notably, this value is significantly lower than the 1/7554 value estimated by Hannah et al., [12] on the basis of gnomAD v2.0.2 data [12]. We propose three possible reasons for this discrepancy. First, updates in the gene list and its accuracy: This study included 31 “Definitive” or “Strong” associated genes as per the latest ClinGen evaluation, whereas Hannah et al., analyzed 29 genes, which may have influenced the overall frequency. Second, database version iteration and sample characteristics: the gnomAD v4.1.0 database used in this study, with a sample size exceeding 800,000, far surpasses the v2.0.2 version used by Hannah et al., (Approximately 180,000). Moreover, gnomAD’s subsequent version update strategy more strictly excluded individuals with severe childhood-onset diseases and their close relatives during database construction. Since PCD typically manifests with classic symptoms in early childhood [5], the variant frequency in gnomAD v4.1.0 may more closely reflect the baseline level of a “healthy” or “general adult” population, leading to relatively lower estimates of rare pathogenic allele frequencies. Therefore, we speculate that 1/20,103 may more accurately reflect the true lower limit of genetic susceptibility to PCD in the current general population. Third, with respect to differences in variant screening and pathogenicity interpretation criteria, this study followed the ACMG/AMP guidelines and incorporated multiple exclusion criteria, such as lof_flag, lc_lof, LCR, segmental duplication, and non-standard transcripts, to screen pLOF and P/LP missense variants, resulting in the inclusion of 5252 variants. In contrast, Hannah et al., combined the Sherloc classification criteria from the Invitae Laboratory with gnomAD’s pLoF annotations. Different variant screening strategies and pathogenicity interpretation processes inevitably lead to differences in the final variant sets included in calculations. The stricter screening in this study, while improving specificity, may also have resulted in lower frequency estimates because some true pathogenic variants were excluded.

Additionally, this study reaffirms significant ethnic heterogeneity in the genetic susceptibility prevalence of PCD. We observed the highest estimated genetic susceptibility prevalence in Middle Eastern populations (MID) (approximately 1/4744) and the lowest in Finnish populations (FIN) (approximately 1/33628), which is consistent with previous reports of significantly higher PCD prevalence in Arab regions than in other areas [10]. However, as noted in the literature [2], fully disentangling the extent to which such significant interethnic prevalence differences are attributable to true allele frequency differences or influenced by factors such as diagnostic levels, reporting biases, and consanguinity rates in different regions remains challenging. The estimated global carrier frequency of approximately 1/25 indicates that the prevalence of pathogenic variants causing PCD in the population is not as rare as commonly assumed. Particularly in certain specific populations, such as Middle Eastern populations (approximately 1/16) and East Asian populations (approximately 1/19), the carrier frequency has reached a relatively high level, approaching the carrier rates of certain common monogenic disorders, such as thalassemia, in specific regions [20]. This carrier frequency, comparable to that of conditions such as spinal muscular atrophy in certain populations (carrier frequency ~ 1/40–1/60 in some ethnic groups), underscores the potential utility of including PCD genes in expanded carrier screening panels, particularly for consanguineous couples or high-risk ethnic groups such as Middle Eastern and East Asian populations, where carrier frequencies approach 1/16 and 1/19, respectively. This disparity is reflected in genetic susceptibility prevalence and carrier frequency and the contribution of pathogenic genes. With respect to pathogenic gene contributions, *DNAH9* gene variants were the most common in the overall population, accounting for approximately 35%, followed by *DNAH5* (approximately 25%) and *DNAH11* (approximately 18%). These gene contribution patterns are largely consistent with previous reports based on clinical cohorts [6]. In East Asian populations (EAs), *ODAD4* gene variants contributed 28%, surpassing *DNAH9* (approximately 20%) and *DNAH5* (13%), whereas in Middle Eastern populations (MIDs), *DNAH5* gene variants were the most common, accounting for 35%, followed by *DNAAF1* (30%). The high carrier rate of PCD revealed in this study and its significant ethnic heterogeneity suggest precise genetic counseling and offspring risk assessment, particularly in populations with extremely high carrier rates (e.g., Middle Eastern populations, approximately 1/16). Exploring and implementing expanded carrier screening in specific high-risk populations can also help guide marriage and reproductive decisions, especially in regions with a high incidence of consanguineous marriages, thereby promoting more comprehensive genetic evaluation of high-risk families and achieving effective primary prevention of the disease.

Comparing our estimates with clinical registry data provides important context. Clinical incidence rates from national registries vary considerably: Primary ciliary dyskinesia (PCD) has an estimated incidence of 1/15,000–1/30,000 live births by Orphanet (https://www.orpha.net/), but this is probably underestimation. Prevalence is difficult to determine. Notably, the lower end of these clinical estimates (~ 1/20,000) aligns closely with our minimal genetic prevalence estimate (1/20,103), while the higher end (~ 1/10,000) is approximately twice our estimate. This alignment suggests that our minimal genetic prevalence may represent a reasonable lower bound for true clinical prevalence, and that the higher clinical estimates may reflect more complete ascertainment in certain populations or inclusion of milder cases that might not have been captured by strict genetic criteria. The discrepancy between our estimate and the 1/10,000 figure also highlights that current genetic testing identifies only ~ 65–70% of cases, and that some clinically diagnosed patients may have pathogenic variants in as-yet-unidentified genes or noncoding regions not captured by our analysis.

This study’s primary strength lies in the use of the latest and largest available gnomAD v4.1.0 database, combined with the latest ClinGen expert panel evaluations of PCD pathogenic genes and a systematic variant screening process. However, several limitations warrant careful consideration. First, population representational bias: Despite gnomAD’s large scale, its population composition remains biased. Non-European populations, particularly Asian and African populations, are underrepresented relative to their actual global population proportions. This bias may limit the direct extrapolation of the results of this study globally and adversely affect the accuracy of genetic susceptibility prevalence estimates in specific underrepresented populations. Second, there are limitations in understanding the genetic basis of PCD: the current knowledge of the genetic basis of PCD is incomplete, and undiscovered or unanalyzed pathogenic genes may still exist. Therefore, the 65% gene diagnostic rate correction factor used in this study is an estimate based on current knowledge, and its accuracy directly impacts the final prevalence estimate. Future discoveries of new pathogenic genes will necessitate adjustments to this correction factor. Third, inherent challenges in variant interpretation and detection technology exist: This study relies on current variant annotation databases and pathogenicity classification criteria. Accurate understanding of a large number of variants of uncertain significance (VUS) remains a significant challenge. Additionally, standard short-read sequencing workflows have limited capture efficiency for certain types of genetic variants (e.g., large copy number variations (CNVs), deep intronic variants, complex structural rearrangements, and variants within specific repeat regions), and these inadequately detected variants may lead to underestimation of prevalence. Fourth, with respect to the Hardy‒Weinberg equilibrium assumption, the calculation of genetic susceptibility prevalence is based on the assumption that the population is in Hardy‒Weinberg equilibrium. However, real populations may deviate from this ideal state because of selection, mutation, migration, and nonrandom mating (e.g., consanguinity), potentially affecting the accuracy of the estimates.

Currently, owing to the complex pathophysiological mechanisms and polygenic inheritance patterns of PCD, the clinical diagnosis and treatment of this disease still face challenges. However, significant progress has been made in diagnosis. The 2018 ATS PCD diagnostic guidelines [25] historically recommended expanding gene panels as a core diagnostic tool alongside nasal NO measurement, anticipating that diagnostic sensitivity would improve with technological advancements. Our study, which analyzed the largest population database to date, revealed significant heterogeneity in the contribution of PCD pathogenic genes among different ethnic groups globally. This finding provides an optimization direction for future genetic diagnosis. These findings highlight that, while comprehensive gene sequencing remains the diagnostic gold standard, understanding population-specific variant profiles can inform the interpretation of genetic testing results and guide counseling, particularly in regions with high prevalence or consanguinity, though caution is warranted in admixed populations where ethnicity-based assumptions may lead to missed diagnoses. As the understanding of the PCD genetic map continues to improve, the diagnostic rate of genetic testing will gradually increase, further strengthening its core position in the PCD diagnostic process, especially when nNO testing is limited by equipment, cost, or patient age. Its core position as a first-line diagnostic tool will become increasingly prominent, ultimately achieving faster and more accurate diagnoses.

At the treatment level, progress in diagnosis further highlights the current lack of treatment options. There is no cure for PCD, and the current PCD treatment guidelines largely still draw from diseases such as cystic fibrosis (CF), lacking high-level evidence specific to PCD [7]. Clinical trials have improved airway mucus clearance by blocking the epithelial sodium channel (ENaC [24] and reducing the frequency of acute respiratory exacerbations in PCD patients treated with azithromycin maintenance therapy [16]. Both approaches focus on preventing and managing complications rather than targeting the underlying cause^30^. The results of these two studies collectively suggest that, given the complex pathophysiological mechanisms of PCD and the profound genetic heterogeneity revealed by this study, future strategies may need to adopt multitarget combinations or more precise individualized diagnosis and treatment strategies. With the rapid development of genetic diagnostic technology, the diagnostic rate of PCD is expected to continue to improve, which will lay the foundation for achieving individualized treatment on the basis of precise molecular typing (such as gene therapy, mRNA transcript therapy, or nonsense mutation readthrough therapy) [22].

In summary, the PCD genetic susceptibility prevalence estimate provided by this study, which is based on gnomAD v4.1.0 (approximately 1/20103 globally), offers the latest reference data for understanding the disease burden of this rare disease. The results highlight the necessity and value of continuously updated large-scale genomic databases for rare disease epidemiological research, as database iterations significantly impact prevalence estimates. The observed ethnic heterogeneity is highly important for guiding genetic counseling and diagnostic strategies for specific populations. In the future, with the release and integration of more diverse population databases (such as NyuWa and ChinaMAP), the discovery of new pathogenic genes, and a deeper understanding of variant functions, we will be able to more accurately depict the genetic map of PCD in the global population, enrich the knowledge of PCD disease characteristics and treatment bottlenecks, and continue to explore more effective interventions to address this rare disease driven by complex genetic factors.

## Supplementary Information

Below is the link to the electronic supplementary material.

Supplementary Material 1: Supplementary Figure 1. Stepwise filtering process for variant inclusion in the genetic susceptibility prevalence calculation

Supplementary Material 2: Supplementary Table 1. Sensitivity analysis of adjusted global and population-specific prevalence estimates under alternative diagnostic yield assumptions

## Data Availability

The data that support the findings of this study are openly available in the Genome Aggregation Database (gnomAD) at https://gnomad.broadinstitute.org/. The specific gene list was derived from the ClinGen Motile Ciliopathy Gene Curation Expert Panel recommendations at https://clinicalgenome.org/, which are also publicly accessible.
